# What Tinnitus Therapy Outcome Measures Are Important for Patients?– A Discrete Choice Experiment

**DOI:** 10.3389/fneur.2021.668880

**Published:** 2021-05-25

**Authors:** Maaike M. Rademaker, Brigitte A. B. Essers, Robert J. Stokroos, Adriana L. Smit, Inge Stegeman

**Affiliations:** ^1^Department of Otorhinolaryngology, Head and Neck Surgery, University Medical Center Utrecht, Utrecht, Netherlands; ^2^University Medical Center Utrecht Brain Center, University Medical Center Utrecht, Utrecht, Netherlands; ^3^Department of Clinical Epidemiology and Medical Technology Assessment (KEMTA), Maastricht University Medical Centre, Maastricht, Netherlands; ^4^Department of Ophthalmology, University Medical Center Utrecht, Utrecht, Netherlands; ^5^Epidemiology and Data Science, Amsterdam University Medical Centers, University of Amsterdam, Amsterdam, Netherlands

**Keywords:** tinnitus, discrete choice experiment, choice, outcome measures, patient's preference, treatment

## Abstract

**Introduction:** The therapeutic rationale varies among tinnitus therapies. A recent study identified which outcome measures should be used for different types of interventions. What patients consider the most important outcome measure in tinnitus therapy is unclear.

**Objectives:** To study the preference of the tinnitus patient for different outcome measures in tinnitus therapy.

**Methods:** A discrete choice experiment was conducted. Participants were provided with two alternatives per choice set (nine choice sets total). Each choice-set consisted of four attributes (tinnitus loudness, tinnitus acceptance, quality of sleep and concentration). With a difference in one of three levels (increased, similar or decreased after treatment) between the alternatives. Results were analyzed with a mixed logit model. Preference heterogeneity was explored with covariates, correlating attributes and a latent class analysis.

**Results:** One hundred and twenty-seven participants took part. In the mixed logit models we found that the choice for a tinnitus therapy was significantly affected by all levels of the outcomes, except for a similar level in concentration and tinnitus acceptance. Tinnitus loudness was considered the most important outcome measure relative to the other attributes. Preference heterogeneity was not explained by correlating attributes. The latent class analysis identified two classes. The first class was similar to the mixed logit analysis, except for a non-significance of similar quality of sleep and tinnitus acceptance. The second class showed a statistical significant preference only for increased tinnitus acceptance and similar quality of sleep.

**Conclusion:** Based on this study, tinnitus patients consider loudness the most important outcome measure. However, there is a variance in preference as indicated by the latent class analysis. This study underlines the importance of research into tinnitus heterogeneity. Next, this study highlights the need for research into tinnitus therapies that focus on diminishing tinnitus loudness.

## Introduction

Tinnitus is an experience of sound in the absence of an external stimulus (1). Because of variations in the definitions of tinnitus and differences in the studied population, the reported prevalence numbers vary between 5.1 and 42.7% (2). A cure for tinnitus does not exist at this moment. Treatment is therefore focused on symptom reduction. The European tinnitus guideline recommends Cognitive Behavioral Therapy (CBT) or sound therapies. However, many more treatment options are available, such as but not limited to, pharmacological therapy, Transcranial Magnetic Stimulation (TMS) and complementary therapies (1, 3).

Tinnitus is a heterogeneous condition due to differences in experienced distress, localization and nature of the sound. Also, many different comorbidities are associated with tinnitus, such as anxiety, depression, and sleep- or concentration problems (1, 4, 5). Tinnitus might also have a negative influence on quality of life. Since many different domains can be affected, measuring the impact of tinnitus on daily life and outcomes of treatments focused at these domains is complicated (6).

In 2018 a Delphi study was conducted to explore the core outcome domains for clinical trials in tinnitus interventions (6, 7). After setting definitions of the different potential core outcome measures, agreement was reached among five different types of stakeholders (patients, healthcare professionals, researchers, commercial representatives, funders) to identify nine different outcome measures as most important per different intervention groups (6, 8). For sound therapies tinnitus intrusiveness, ability to ignore, concentration, quality of sleep and sense of control were selected as the core outcome measurements. For psychological based interventions these were intrusiveness, tinnitus acceptance, mood, negative thoughts and beliefs, and sense of control. Finally, for pharmacological based interventions the most important outcome domains were intrusiveness and tinnitus loudness (7). This study highlights the differences in therapeutic approach necessitating different outcomes to be measured. However, this does not tell us what outcome measure is of the most importance for the patient when they seek treatment for tinnitus, and consequently what outcome measure carries the most importance for them?

A discrete choice experiment (DCE) is a quantitative method to elicit preferences from participants. In a DCE, participants are presented with a series of scenarios. Participants are forced to choose between a set of alternatives. This enables us to analyze the most important outcomes for patients who are in need of tinnitus therapy. DCE's are an increasingly popular experiment to asses patient preference in health care (9). For example, it has been previously used to explore preference in colorectal-cancer screening, breast cancer treatment and basal cell carcinoma treatments (10–12). In this study we aim to analyze the preference of patients for outcome measures in tinnitus therapy with a DCE.

## Methods

In a discrete choice experiment (DCE) participants are asked to choose between two or more alternatives within a choice set. Each choice set consists of a set of attributes with different levels. The attributes remain similar while the levels vary over the number of choice sets. The attributes and their corresponding levels are a key element of a DCE.

In this study, tinnitus participants were required to choose between two (hypothetical) tinnitus therapies (alternatives) (treatment A and treatment B). The tinnitus participants were presented with nine different choice sets, each consisted of four attributes with three corresponding levels. An example of a choice-set as used in this DCE is presented in Table 1.

**Table 1:** Example of a choice set.

The attributes (tinnitus loudness, concentration, quality of sleep and acceptation) can have the following levels: - Increased after treatment compared to before treatment (Increase) - Similar relative to before the start of treatment (Similar) - Decreased relative to before the start of treatment (Decrease)		
If both treatments were offered to you, which one would you choose?		
	Option A	Option B
Tinnitus loudness	Decrease	Similar
Concentration	Similar	Increase
Quality of sleep	Similar	Increase
Tinnitus acceptance	Increase	Similar
	□ Option A	□ Option B

The development of this DCE consisted of different stages in chronological order: a focus group to select the most suitable outcomes for inclusion in this DCE, the DCE design, data collection and data analysis.

### Defining Attributes and Levels

#### Focus Groups

For the first step two focus groups with nine participants were organized in July 2019. The focus groups were guided by an interviewer (MR). The aim of the focus group was to gain information on the most important attributes and corresponding levels in order to create a DCE. Participants were instructed to discuss the nine outcomes of the previously conducted Delphi study on concept. The nine outcome measures were tinnitus intrusiveness, ability to ignore, concentration, quality of sleep, sense of control, tinnitus acceptance, mood, negative thoughts and beliefs, tinnitus loudness as defined by the COMIT'ID initiative (supplementary file) (7, 8). The participants of the focus groups had to discuss which of these outcome measures were deemed most suitable to be included in the choice experiment of our study (7). Therefore, these items were introduced verbally to the focus groups, without providing the definitions. They were also explained the concept of the levels, and asked to discuss which were the most suitable. In order to be sure that every participant was actively included in the discussion, we asked an independent researcher (LM) to observe the non-verbal communication of the participants. The observations of non-verbal communication by the independent researcher did not result in extra information about the outcomes of the focus groups. The participants were also asked to rate the nine outcome measures on a five-point Likert-scale (one totally not important to five very important) and list their five most important ones.

The outcomes of the focus group were discussed in the research group (AL, IS, and MR). The results of the focus group were discussed and interpreted to set the final attributes and levels. To end up with a feasible DCE four attributes with different levels were included in the DCE. The five most popular attributes of the focus groups were the attributes tinnitus loudness, concentration, quality of sleep, tinnitus acceptance and tinnitus intrusiveness. After careful deliberation, we decided against including tinnitus intrusiveness, since there is no direct translation of the word in Dutch. Next, the construct of the word tinnitus intrusiveness is difficult and debatable (7). Also, in the focus group we noticed that participants had different understandings of the word intrusiveness. This resulted in the final set of attributes; tinnitus loudness, concentration, quality of sleep and tinnitus acceptance.. The corresponding levels were similar for all attributes: increased after treatment compared to before treatment, similar relative to before the start of treatment, decreased relative to before the start of treatment. Both the attributes and levels were not formally defined in our study. Participants were not provided with a formal definition of the concept. Therefore, participants relied upon their own interpretation.

#### DCE Design

In the next stage, the DCE was created. With four attributes including three levels each, 81 (3^4^) different choice sets can theoretically be created. Since it is not feasible to ask participants to fill out 81 different choice sets, we developed a fractional factorial Bayesian efficient design in Ngene version 1.2.1. 2018. Bayesian efficient designs maximize the information that can be obtained from the choice data and the accuracy of estimate choice model parameters (13, 14).

Thirty-six choice sets, blocked into four versions with each nine different choice sets were created. Participants were randomized in one of four blocks.

An efficient design functions optimally when utility weights or priors of attributes are added to the design. We therefore first conducted a pilot study (*n* = 30) to deduct priors. These priors were subsequently used to update the final design.

#### Questionnaire

Based on above described methodology a questionnaire was developed for participants. The questionnaire consisted of an instruction for the choice-experiment, the choice-sets of the choice experiment, as well as additional questions. The additional questions were used for the baseline characteristics and covariate analysis. They included three questions regarding health literacy, as this could influence outcome of the DCE. Besides this, questions regarding tinnitus characteristics and the impact of tinnitus on daily life (distress) were asked. The questions regarding tinnitus characteristics were based on the tinnitus sample case history questionnaire (TSCHQ) and the ESIT questionnaire (15).

The impact of tinnitus on daily life was measured with the Tinnitus Functional Index (TFI) (16). The TFI is a 25-item questionnaire using 11 point Likert scale questions. The outcome is a score from 0 (not a problem) to 100 (a very big problem). The questionnaire consists of eight subscales; intrusiveness, sense of control, cognition, sleep, hearing, relaxation, quality of life and emotions. In this study we used the validated 2014 Dutch translation of the TFI, with a high reliability as expressed in a Cronbach's alpha of 0.91 (17).

#### Recruitment, Logistics and Ethics

For all steps of the study participants were eligible if they were 18 years or older and sought help or planned to seek help for their tinnitus. For the focus groups participants were recruited from the tinnitus outpatient clinic of the otorhinolaryngology department of the UMC Utrecht by the consulted otologist, from patients visiting a regional audiological clinic and by an announcement on the website of the Dutch tinnitus patients association (*Stichting Hoormij*). These people received information about the study and were subsequently invited to one of two focus groups that took place in the UMC Utrecht. Informed consent was given to use the data collected from the focus groups.

For the pilot DCE and final DCE participants were recruited in the October 2019—march 2020, through an advertisement on the either the website of the Dutch tinnitus patients association *(Stichting Hoormij.nl*) or at the tinnitus outpatient clinic of the UMC Utrecht. The advertisement included a brief summary of the research project. People who applied for study participation were informed about the study procedures by postal/digital mail. When informed consent was obtained and people fulfilled inclusion criteria, participants were included in the study. The questionnaires, including the DCE, were electronically sent to the participants digitally with Castor EDC (18). For the pilot study data was collected in November/December 2019. The final experiment was conducted in February/March 2020. If participants did not respond within one to 2 weeks they were sent a reminder to fill out the questionnaire. The Medical Research Ethics Committee (MREC) of the UMC Utrecht confirmed that the Medical Research Involving Human Subjects Act (WMO) does not apply to this research and an official approval of this study is therefore not required under the WMO (local number 19/690).

#### Sample Size

We estimated a sample size based on the rule of thumb as proposed by Johnson and Orme (19). This is performed with the following formula: *N* > 500c/(t × a). Where t is the number of choice tasks, a the number of alternatives and c the number of analysis cells. However, the calculation of an optimal sample size for estimating non-linear discrete choice models from DCE data is complicated as it depends on the true values of the unknown parameters estimated in choice models (20). Lancsar and Louviere mentioned that based on empirical experience one rarely requires more than 20 respondents per questionnaire version. All information combined led to a minimum sample size requirement of 83 respondents.

#### Data Analysis

In this paper data analysis was performed on the combination of the pilot version and the definitive version of the DCE. Descriptive variables were analyzed with SPSS version 25.0.0.2. Normality was visually assessed. Means and standard deviations (SD) were calculated, just as frequencies. Age was determined as the difference from date of birth to study year. For the pilot group this was 2019, for the final version of the DCE this was 2020.

##### Discrete Choice Data

Data analysis was conducted with the Nlogit econometric software version 6, September 2016. Both a mixed logit and a latent class analysis was applied (21).

##### Mixed Logit Model

A mixed logit model determines the average impact of the different attributes on the utility function. The utility function is expressed as:

$$\begin{array}{l} {\text{U}}_{ij}=\beta_{0}+{\left(\beta_{1}+v_{1i}\right)}^{*}Tinnitus\;loudness\;decrease\text{d}\\ +{\left(\beta_{2}+v_{2i}\right)}^{*}Tinnitus\;loudness\;similar+{\left(\beta_{3}+v_{3i}\right)}^{*}\\ \text{QoS increased }+{\left(\beta_{4}+{\text{v}}_{4\text{i}}\right)}^{*}\text{Tinnitus acceptance}\\ \text{similar}+{\left(\beta_{5}+{\text{v}}_{5\text{i}}\right)}^{*}\text{Tinnitus acceptance increased}\\ +{\left(\beta_{6}+v_{6i}\right)}^{*}Concentration\;similar+{\left(\beta_{7}+v_{7i}\right)}^{*}\\ \text{Concentration increased}+{\left(\beta_{8}+{\text{v}}_{8\text{i}}\right)}^{*}\text{QoS similar}+\varepsilon_{\text{ij}} \end{array}$$

β_0_ is the constant, β_1_ to β_8_ are the mean attribute utility weights and v1i to v8i are errors, which describe individual variation to the utility weights. **ε**_**ij**_ is an error part.

All variables were effect coded. “With effects coding, all nonomitted levels are coded as −1 when the omitted level is present. The coefficient on the omitted level of an effects-coded variable can be recovered as the negative sum of the coefficients on the nonomitted levels of that attribute. Therefore, effects coding yields a unique coefficient for each attribute level included in the study” (22)^p303^. Reference levels were the worst potential outcome; i.e., increased tinnitus loudness and decreased concentration, sleep quality and tinnitus acceptance. The mixed logit model allows for variation around preferences in the population. The preferences are described with a β (mean) and a standard deviation (SD) of the error term. A positive or negative sign indicates the attribute level is either preferred or not preferred.

In our model, random parameters were defined by a normal distribution using halton draws with 500 repeated simulations. At first, all attributes were defined as random parameters. Attributes without a statistically significant standard deviation were no longer defined as random parameters in the next model (with a smaller set of random parameters and the other parameters as fixed). To explore preference heterogeneity covariates (age, tinnitus distress and gender) were added as interactions to the model. Only statistically significant interactions were kept in the final model. Best model fit was based on the log likelihood function.

A ranking in relative importance was calculated by dividing the random parameter's utilities range between the worst and best level by the total sum of all parameters.

##### Latent Class Model

To further analyze preference heterogeneity a latent class analysis (LCLOGIT) was performed with different amount of classes (two to seven). Best model fit was based upon the Aikake information criterium (AIC), the AIC/N and clinical interpretability/relevance. Since the classes are “latent,” it is not known which participants belongs to which class. However, by means of posterior probabilities we made the best estimate to which class a participants belongs (23). This information was used to describe the classes with the baseline characteristics.

## Results

There were 127 participants in our study. Thirty out of thirty (100%) participated in the pilot version. Ninety-seven of 98 participants (99%) who signed informed consent filled out the definitive version. In this study data of the pilot version and the final version are reported. The mean age of the respondents of both the pilot and final version was 62.2 years of age (SD 10.3). 54 of 127 (42.5%) participants were female and the mean TFI score was 45.2 (SD 20.1) (Table 2). Considering health literacy 106 out of 127 participants (83.5%) never needed help with reading information from the hospital or general practitioner. Ninety of 127 (70.9%) were very much certain that they filled out medical forms correctly themselves and 93 of 127 (73.2%) did not experience difficulties with written information (Table 3).

**Table 2:** Baseline characteristics of study participants for total study group and split per different classes (based on Table 5).

**Characteristic**		**Total study population**	**Classes**	
		**(*n* = 127)**	**Class 1 (*n* = 72)**	**Class 2 (*n* = 55)**
		***N* (%)**	***N* (%)**	***N* (%)**
Age (years)^a^		62.2 (10.3)	62.0 (10.3)	62.5 (10.4)
Gender (female)		54 (42.5)	33 (45.8)	21 (38.2)
TFI ^a^		45.2 (20.1)	46.5 (20.6)	43.5 (19.5)
**TFI subscales^a^**				
Intrusiveness		60.2 (22.7)	62.4 (22.7)	57.3 (22.5)
Sense of control		61.2 (20.1)	62.9 (20.6)	58.9 (19.4)
Cognitive		36.7 (23.4)	38.8 (24.3)	33.9 (22.1)
Sleep		43.4 (31.5)	42.3 (32.4)	44.8 (30.5)
Auditory		45.5 (29.0)	42.9 (28.9)	48.8 (29.0)
Relaxation		44.2 (26.3)	45.9 (27.3)	42.0 (24.9)
Quality of life		37.5 (26.7)	39.8 (27.5)	34.5 (25.6)
Emotional		35.6 (26.8)	39.6 (27.7)	30.4 (24.8)
**Scales 1–10^a^**				
Acceptance		6.4 (2.2)	6.2 (2.3)	6.6 (2.0)
Loudness		6.7 (2.1)	6.5 (2.2)	6.9 (1.9)
Concentration		5.3 (2.1)	5.4 (2.1)	5.3 (2.2)
**Tinnitus characteristics**				
Start of tinnitus	Less than 3 months ago	0 (0.0)	0 (0.0)	0 (0.0)
	3–6 months ago	5 (3.9)	3 (4.2)	2 (3.6)
	6 months or longer	122 (96.1)	69 (95.8)	53 (96.4)
Pattern	Constant	114 (89.8)	64 (88.9)	50 (90.9)
	Intermittent	13 (10.2)	8 (11.1)	5 (9.1)
Number of sounds	One	67 (52.8)	41 (56.9)	26 (47.3)
	More than one	60 (47.2)	31 (43.1)	29 (52.7)
	Amount^a^	3.0 (1.4)	3.1 (1.4)	3.1 (1.4)
Pulsatile	Yes	20 (15.7)	11 (15.3)	9 (16.4)
Hearing difficulties	I hear nothing	2 (1.6)	1 (1.4)	1 (1.8)
	Severe problems	42 (33.1)	21 (29.2)	21 (38.2)
	Mediocre problems	37 (29.1)	19 (26.4)	18 (32.7)
	Small problem	29 (22.8)	18 (25.0)	11 (20.0)
	No problem	17 (13.4)	13 (18.1)	4 (7.3)
Sought help		120 (94.5)	69 (95.8)	51 (92.7)
Type of help	Self-management	85 (70.8)	48 (69.6)	37 (72.5)
	Psychological treatment	67 (55.8)	41 (59.4)	26 (51.0)
	Audiological treatment	63 (52.5)	36 (52.2)	27 (52.9)
	Physiotherapy	28 (23.3)	21 (30.4)	7 (13.7)
	Psychiatric treatment	20 (16.7)	11 (15.9)	9 (17.6)
	Alternative treatment	50 (41.7)	33 (47.8)	17 (33.3)
	Other	13 (10.8)	7 (10.1)	6 (11.8)
Plans to seek help		6 (4.7)^*^	2 (2.8)	4 (7.3)
Type of help	Self-management	3 (50.0)	1 (50.0)	2 (50.0)
	Psychological treatment	4 (67.0)	1 (50.0)	3 (75.0)
	Audiological treatment	4 (67.0)	1 (50.0)	3 (75.0)
	Physiotherapy	1 (16.7)	0 (0.0)	1 (25.0)
	Psychiatric treatment	0 (0.0)	0 (0.0)	0 (0.0)
	Alternative treatment	2 (33.3)	1 (50.0)	1 (25.0)
	Other	1 (16.7)	0 (0.0)	1 (25.0)

a *Mean (standard deviation)*.

Other–sought help: Neuromodulation, earplugs, none (n = 5)**, doctor, ghnatologist, orthomanual therapist, supplements, EMDR, electromagnetic pulses. Plans to seek help: implants.

* *1 person answered both questions did you seek help or do you plan to seek help negatively. However, since the participant answered positively at the question at inclusions, the data was included in the analyses*.

^**^The same was applicable for the 5 people that answered in the open area box: none. They however did answer positively at the question did you seek help.

**Table 3:** Health Literacy questions and outcome.

**Health literacy**		***N* (%)**
How often does somebody help you with reading letters or folders from your general practitioner or the hospital?		
	Never	106 (83.5)
	Occasionally	18 (14.2)
	Sometimes	0 (0.0)
	Often	2 (1.6)
	Always	1 (0.8)
How certain are you that you fill out medical forms correctly yourself?		
	Very much	90 (70.9)
	Quite	32 (25.2)
	A little	1 (0.8)
	A very little	1 (0.8)
	Not at all	3 (2.4)
How often is it difficult for you to understand more about your health, because you do not completely understand written information?		
	Never	93 (73.2)
	Occasionally	26 (20.5)
	Sometimes	7 (5.5)
	Often	0 (0.0)
	Always	1 (0.8)

### Preferences

The main results of choice experiment by the mixed logit model are presented in Table 4. The final model had a log-likelihood function of −587.77 and an adjusted pseudo *R*^2^ of 0.258. Uniform distributions were tested, but did not improve the model. All variables presented are main effects.

**Table 4:** Result of the mixed effect model.

	**Main model**			**Model including covariates**		
**Attributes and levels**	**Estimate (95% CI)**	**Standard deviation (95% CI)**	**Relative Importance**	**Estimate (95% CI)**	**Standard deviation (95% CI)**	**Relative importance**
**Random parameters in utility function**						
Tinnitus loudness decreased^a^	2.03 (1.48–2.58)^***^	2.00 (1.46–2.53)^***^	0.47 (1)	2.03 (1.48–2.59)^***^	1.98 (1.46–2.51)^***^	0.44 (1)
Tinnitus loudness similar	0.31 (0.11–0.50)^***^	0.45 (0.14–0.76)^***^		0.31 (0.12–0.50)^***^	0.44 (0.14–0.74)^***^	
QoS increased	0.88 (0.57–1.18)^***^	0.95 (0.60–1.31)^***^	0.19 (3)	0.88 (0.57–1.19)^***^	0.95 (0.59–1.31)^***^	0.21 (3)
Tinnitus acceptance similar	0.25 (0.05–0.44)^**^	0.46 (0.18–0.74)^***^		0.72 (0.28–1.16)^***^	0.43 (0.14–0.72)^***^	
Tinnitus acceptance increased	0.90 (0.65–1.15)^***^	0.60 (0.32–0.87)^***^	0.22 (2)	0.90 (0.65–1.44)^***^	0.61 (0.33–0.88)^***^	0.25 (2)
**Fixed parameters**					
Constant	−0.12 (−0.33–0.08)			−0.13 (−0.33–0.08)		
Concentration similar	0.001 (−0.161–0.164)		0.11 (4)	0.001 (−0.16–0.16)		0.10 (4)
Concentration increased	0.51 (0.30–0.72)^***^			0.50 (0.29–0.71)^***^		
QoS similar	0.38 (0.20–0.56)^***^			0.38 (0.20–0.56)^***^		
(Interaction Tinnitus acceptance similar x TFI)				−0.01 (−0.02 – −0.002)^**^		
LogLikelihood	−587.77			−584.86		
Chi squared	(14) 408.9 (*p* = 0.000)			(15) 414.8 (*p* = 0.000)		
Adjusted pseudo R^2^	0.258			0.262		
AIC/N	1.053			1.050		

a *Effect coding: worst level can be retrieved for example increased tinnitus loudness: −1.0 (2.03+0.31) = −2.39,*

*** *significance at 1% level,*

** *significance at 5% level*.

Respondents showed a significant preference for a tinnitus treatment that results in a decrease [β = 2.03 (1.48–2.58)] or similar level tinnitus loudness [(β= 0.31 (0.11–0.50)], an increase in [β = 0.88 (0.57–1.18)] or similar level of quality of sleep [β = 0.38 (0.20–0.56)], an increased [β = 0.90 (0.65–1.15)], or similar tinnitus acceptance [β = 0.25 (0.05–0.44)] and an increase in concentration [β = 0.51 (0.30–0.72)]. Overall, the choice for a tinnitus therapy was significantly affected by all levels of the outcomes, except for a similar level in concentration. In addition, all signs are in the expected direction (positive β's), confirming theoretical validity of the model.

All standard deviations of the random parameters were statistically significant, indicating preference variation among participants. To explore the heterogeneity, three covariates (age, gender and TFI score) were added to the model. A significant interaction was found with a similar level of tinnitus acceptance and the TFI of β = −0.01 (−0.02 to −0.001). Adding this interaction improved the model significantly to a LL of −584.86, with an adjusted pseudo *R*^2^ of 0.26. The interaction changed the level of significance of the main effect of similar tinnitus acceptance from 5 to 1% [β = 0.72 (0.28–1.16)]. Correlations among all different parameters were explored; the model did not improve significantly and was therefore not reported.

The relative importance of the random parameters was calculated for both the main effect model and the model with the interaction. Similar results were yielded. Tinnitus loudness was the most important outcome measure, followed by tinnitus acceptance, quality of sleep and concentration in that order.

### Attribute Trade-off

By inserting parameter estimates and attribute levels in the utility function, we gain insight in how participants were willing to trade of between levels of attributes. For example, a change from decreased tinnitus loudness (β = 2.03) to similar tinnitus loudness (β = 0.31) would lead to a utility decrease of Δ −1.72, when all other attributes would remain similar. An increase in tinnitus acceptance (Δ +0.65), quality of sleep (Δ +0.50) and concentration (Δ +0.52) from the similar level would lead to a utility increase of Δ +1.67. Since 1.67 is smaller than 1.72, this utility increase does not compensate the utility decrease of tinnitus loudness.

### Latent Class Analysis

Models were made for two to seven different classes. The choice for optimal latent class model was based on model fit and clinical interpretability. Only the model with two classes could be interpreted clinically. The model showed an AIC of 1247.4 and an AIC/N of 1.091. The first class had an estimated latent class probability of 0.57 (0.44–0.70), the second of 0.43 (0.30–0.56). The first class was similar to the mixed logit model in terms of significant parameters, except for an insignificant similar level of QoS and tinnitus acceptance in the first class [β = −0.03 (−0.33–0.27), −0.06 (−0.33–0.21)]. Tinnitus loudness was still considered the most important attribute relative to sleep, tinnitus acceptance and concentration in that order. In the second model statistical significance was achieved for two attributes; a similar level of QoS (β = 0.31 (0.12–0.50) and an increased level of tinnitus acceptance [β = 0.51 (0.32–0.70)]. Tinnitus acceptance was the most important attribute relative to sleep, concentration and tinnitus loudness in that order (Table 5).

**Table 5:** Outcome of the latent class analysis.

	**Class 1**		**Class 2**	
	**Estimate (95% CI)**	**Relative importance**	**Estimate (95% CI)**	**Relative importance**
Tinnitus loudness decreased	2.65 (1.98–3.31)^***^	0.56 (1)	−0.01 (−0.34–0.31)	0.02 (4)
Tinnitus loudness similar	0.48 (0.23–0.73)^***^		−0.04 (−0.23–0.15)	
Concentration similar	0.02 (−0.18–0.22)		−0.002 (−0.17–0.16)	
Concentration increased	0.58 (0.32–0.83)^***^	0.11 (4)	0.19 (−0.01–0.40)	0.16 (3)
QoS similar	−0.03 (−0.33–0.27)		0.31 (0.12–0.50)^***^	
QoS increased	0.95 (0.66–1.23)^***^	0.18 (2)	0.24 (−0.02–0.50)	0.33 (2)
Tinnitus acceptance similar	−0.06 (−0.33–0.21)		0.17 (−0.02–0.37)	
Tinnitus acceptance increased	0.76 (0.50–1.02)^***^	0.14 (3)	0.51 (0.32–0.70)^***^	0.49 (1)
Estimated latent class probabilities	0.57 (0.44–0.70)		0.43 (0.30–0.56)	
Aikakes information criterium	1247.4			
AIC/N	1.091			

*** *Significance at 1% level*.

The mean age was 62.0 (SD 10.3) for class 1 and 62.5 (SD 10.4) for class 2. The mean TFI score was 46.5 (SD 20.6) for class 1 and 43.5 (SD 19.5) for class 2. Class 1 had a mean of 42.3 (SD 32.4) on the TFI subscale sleep, compared to 44.8 (30.5) in class 2. Class 1 scored a mean score of 6.2 (SD 2.3) on the VAS scale for acceptance, 6.5 (SD 2.2) on loudness and 5.4 (2.1) on concentration, compared to 6.6 (SD 2.0) for acceptance, 6.9 (SD 1.9) on loudness and 5.3 (SD 2.2) for concentration in class 2. 31 of 72 (43.1%) participants in class 1 experienced more than one sound, compared to 29 of 55 (52.7%) participants of class 2.

## Discussion

In this study we conducted a discrete-choice experiment to understand the preference of tinnitus patients for outcome measures in tinnitus therapy. In a mixed logit analysis we found that a decrease in tinnitus loudness was the most important outcome measure compared to the others. A change from decreased tinnitus loudness to a similar level of tinnitus loudness, could not be compensated by an increase in levels for the other three attributes (sleep, concentration and tinnitus acceptance). Preference heterogeneity was present, since all standard deviations of the random parameters were statistically significant in the mixed logit model. Preference heterogeneity could not be explained by correlating the attributes, but there was a significant model improvement with the interaction of similar level of tinnitus acceptance and the TFI. The optimal model of the latent class analysis showed two classes. The first class was very similar to the mixed logit analysis; primarily a decrease of tinnitus loudness was preferred next to an increase of the other attributes. In the second class only an increase in tinnitus acceptance or a similar level of quality of sleep was preferred. The mean TFI score of 45.2 (SD 20.1) can be interpreted as that tinnitus is considered a moderate problem by the participants according to the grading of the TFI (16). This is in correspondence with our inclusion criteria that participants were in need or have been in need of help.

Tinnitus loudness was considered the most desirable outcome compared to the other attributes. This means that tinnitus loudness is the most desirable outcome measure for tinnitus patients in treatment relative to quality of sleep, tinnitus acceptance and concentration. Assessing tinnitus loudness however, has its difficulties. First there is no consensus of one standardized test for measuring tinnitus loudness (24). For example, the perceptual attributes can be measured with tinnitus matching experiments (25, 26). The subjective impact of loudness can be measured with self-reported scales (27). Discrepancies have been described between subjective and objective measures (28, 29). These discrepancies demonstrate the difficulties in the concept of tinnitus. Even though the description of the phenomenon tinnitus is straightforward, the concept of what it means for patients varies greatly (30, 31). Loudness alone does not fully explain the experienced distress and therefore, a decrease in subjective loudness does not necessarily correlate with a similar amount of decrease in tinnitus distress. This is in accordance with tinnitus distress models where tinnitus distress encompasses emotion and reaction next to the sound experience (29, 32–34). This idea is also grasped in the TFI. The total score consists of eight different domains that could all have an effect on the total impact of tinnitus on daily life (16).

The outcomes of this study raise the question on how to reduce the tinnitus loudness. A systematic review showed that most trials that aim to reduce tinnitus loudness are pharmacological trials (25). A previous study on preferences on outcomes in tinnitus patients showed that 52% were very interested to take a pill if it would reduce tinnitus loudness and annoyance by half. 62% would even take a pill if the tinnitus loudness and annoyance would be completely eliminated (35).

The latent class analysis showed that 57% of the participants considered an improvement in all attributes important. They have the strongest preference for tinnitus loudness, relative to quality of sleep, tinnitus acceptance and concentration in that order, similar to the mixed logit model. However, for 43% of the participants tinnitus acceptance and sleep were the most important outcome measures. Both classes were very similar based on the baseline characteristics. They had similar mean scores on the total TFI and the TFI subscale on sleep. The same is applicable for the VAS-scales on tinnitus acceptance, loudness and concentration. Even though the first class prefers a loudness and the second class acceptance. Differences can be found on the amount of experienced sounds; class 2 seemed to experience more sounds. One might hypothesize that a higher total amount of sounds might explain that an increase in QoS and acceptance is preferred over a decrease in loudness. However, this is not explained by the similar levels on the VAS scale for acceptance and the TFI subscale for sleep. Please note, as stated in the methods section, these are estimates of belongings to classes. Since these are “latent” classes, the true belonging of an individual to a class cannot be assessed (23).

Heterogeneity in tinnitus complaints is a common issue in tinnitus research, and limits the generalizability of therapy outcomes that might focus on one aspect of this disease (4, 36). It is commonly believed that there are subtypes of tinnitus patients (4, 36). Therefore, this study stresses the need for research of finding these subtypes of tinnitus patients which could be related to the preferred outcome measure for tinnitus therapy. Next this study underlines the importance of shared decision making in the process of choosing suitable therapy.

The lack of adequate and evidence based treatments for different tinnitus patients highlights the importance of improving the methods for tinnitus research (36). This starts with defining outcomes, defining the exact study population and patient's needs (6, 37). The heterogeneity of the condition and its patients makes it challenging to define criteria for reliable and effective treatment trials. We believe that defining the preference of patients, could function as a foundation for defining outcomes (7). Additionally it provides insight in the heterogeneity and subtypes of patients affected by the condition. The COMiT'ID study focused on uniformity of research and developed a core outcome set for tinnitus research. The authors recommend specific outcome measures for different intervention types. For example tinnitus loudness should be an outcome measure in drug therapies (7). In this study we solely assessed the choices people make in a selection of outcome measures aimed at treatment, independent of intervention type. The combination of both studies could be of importance for future trials. Based on that perspective both the Delphi trial and this discrete choice experiment could be complementary to each other (6). Next, we recommend more research into therapies that might diminish tinnitus loudness, not necessarily only drug therapies. We encourage authors to consider loudness to be assessed as an additional outcome measure to the core set in the other intervention types (sound, psychological) as recommend by the COMiT'ID.

### Strengths and Limitations

There are several limitations applicable to this study. The primary limitation is the lack of a formal definition of the attributes and levels. The outcome measures used in this study, were previously defined in the COMiT'ID studies as follows: “*tinnitus loudness: how loud your tinnitus sounds, quality of sleep: getting the right amount of undisturbed sleep for you that leaves you feeling refreshed and rested, tinnitus acceptance: recognizing that tinnitus is a part of your life without having a negative reaction to it, concentration: ability to keep your attention focused”(**8**)*^additional file^. Participants in our study were not instructed with any formal definitions. They had to rely upon their own interpretation. Participants could have had different ideas and concepts for the different attributes and levels used in this study. The second is the fact that only a small set of (four) attributes could be investigated in order to make the DCE feasible. We acknowledge that the participants might prefer other outcome measures outside of the pre-selected outcome measures of this study (e.g., the effect of tinnitus on hearing). Also, the attributes were based on a previously conducted elaborate Delphi experiment. However the selection of the outcome measures for our study was based on discussion in the focus groups and the research group (7). Another limitation of this study was that it did not include the specific type of intervention. It might be interesting to observe what will happen if intervention type would be added as an attribute or in a labeled design. A fourth limitation is the fact that we included participants only if they planned to seek help for their tinnitus or if they had already sought help. A bias could have been introduced by participants that did not have an active wish (anymore) for help at the moment of filling out the questionnaire.

### Conclusions

A discrete choice experiment was conducted in order to understand the preference of tinnitus patients in four different outcome measures (tinnitus loudness, tinnitus acceptance, quality of sleep, and concentration) for tinnitus therapy. The experiment forced participants to choose the most important attribute with a specific level. A decrease in tinnitus loudness was considered the most important outcome measure compared to quality of sleep, tinnitus acceptance and concentration. The mixed logit analysis showed heterogeneity that was not explained by covariates. A statistically significant interaction was found between a similar level and tinnitus acceptance and the TFI score. A latent class analysis showed two classes. The first class was similar to results of the mixed logit analysis, the second showed a statistical significant preference only for tinnitus acceptance and quality of sleep. This study stresses the importance of researching tinnitus heterogeneity. Also, this study highlights the need for research into tinnitus therapies that might diminish tinnitus loudness.

## Data Availability Statement

The datasets presented in this article are not readily available because of legal issues. Requests to access the datasets should be directed to the corresponding author.

## Ethics Statement

Ethical review and approval was not required for the study on human participants in accordance with the local legislation and institutional requirements. The patients/participants provided their written informed consent to participate in this study.

## Author Contributions

MR, AS, and IS contributed to data collection. MR, BE, and IS contributed to the methodology and statistical analyses. MR wrote the first version of the manuscript. All authors contributed to manuscript revision, read, approved the submitted version, contributed to the conception, and the design of the study.

## Conflict of Interest

The authors declare that the research was conducted in the absence of any commercial or financial relationships that could be construed as a potential conflict of interest.
