# Supplementary material for: What Tinnitus Therapy Outcome Measures Are Important for Patients?– A Discrete Choice Experiment
Source: Front Neurol. 2021 May 25;12:668880. doi: 10.3389/fneur.2021.668880 (PMC8185356; doi:10.3389/fneur.2021.668880)
Supplement: Supplementary file 1 [file Table_1.DOCX]

**Supplementary file.**

**Definitions of outcome measures as defined by the COMIT’ID initiative.**(1)

**Please note:** we directly copied all the definitions in the table below from the additional file of

*Fackrell K, Smith H, Colley V, Thacker B, Horobin A, Haider HF, et al. Core Outcome Domains*

*for early phase clinical trials of sound-, psychology-, and pharmacology-based interventions*

*to manage chronic subjective tinnitus in adults: The COMIT’ID study protocol for using a*

*Delphi process and face-to-face meetings to establis. Trials. 2017;18(1):1–11.*

| **Outcome measure** | **Definition** |
| --- | --- |
| Ability to ignore | Ability to continue as normal as if tinnitus were not there |
| Concentration | Ability to keep your attention focused |
| Acceptance of tinnitus | Recognizing that tinnitus is a part of your life without having a negative reaction to it |
| Mood | General sense of well-being, ranging from feeling very low or negative to very positive |
| Negative thoughts/beliefs | Thinking tinnitus will affect you in a negative way (e.g. thinking that tinnitus is never going to get better or that It would be dreadful if these noises never went away) |
| Tinnitus intrusiveness | Noticing the sound of tinnitus is there and it is invading your life or your personal space |
| Tinnitus loudness | How loud your tinnitus sounds |
| Quality of sleep | Getting the right amount of undisturbed sleep for you that leaves you feeling refreshed and rested |
| Sense of control | Whether or not you feel you have a choice in how to manage the impact of tinnitus and feelings caused by tinnitus |

Reference:

1. Fackrell K, Smith H, Colley V, Thacker B, Horobin A, Haider HF, et al. Core Outcome Domains for early phase clinical trials of sound-, psychology-, and pharmacology-based interventions to manage chronic subjective tinnitus in adults: The COMIT’ID study protocol for using a Delphi process and face-to-face meetings to establis. Trials. 2017;18(1):1–11.
